# Stochastic Dynamics of Sensitive and Resistant Bacteria Under Capacity-Limited Antibiotic Treatment

**DOI:** 10.3390/e28101107

**Published:** 2026-10-11

**Authors:** A. Almutairi, M. A. Sohaly

**Affiliations:** 1 Department of Mathematics, College of Science, Qassim University, Buraydah 51452, Saudi Arabia; almutairi2017@qu.edu.sa; 2 Department of Statistics and Operations Research, College of Science, Qassim University, Buraydah 51452, Saudi Arabia

**Keywords:** antimicrobial resistance, Markov population process, mutation-selection dynamics, extinction, mathematical models simulation, bifurcation analysis

## Abstract

Antimicrobial resistance is a major challenge in the effective treatment of bacterial infections because antibiotic exposure can selectively suppress sensitive bacteria while allowing resistant populations to persist and expand. In this study, we develop a finite-state stochastic population model for the joint dynamics of antibiotic-sensitive and antibiotic-resistant bacteria under capacity-limited treatment. The model is formulated as a bivariate continuous-time Markov chain with density-dependent logistic growth, sensitive-to-resistant mutation, differential antibiotic susceptibility, finite carrying capacity, and bounded treatment-removal capacity. Because the extinction state is absorbing, the ordinary stationary distribution of the full finite-state process is concentrated at extinction, and long-lived non-extinct behavior is therefore analyzed through transient and quasi-stationary perspectives. Exact first-moment equations are derived, showing that mutation transfers individuals from the sensitive population to the resistant population without creating additional bacterial individuals. Local stability near extinction is characterized by the growth rates $r_{s}=\lambda_{s}-\mu -\nu$ and $r_{r}=\lambda_{r}-\epsilon \mu$. The positive equilibria of the deterministic fluid approximation are then derived under the different treatment-capacity regimes, while capacity-induced critical transitions are characterized through the equilibrium equations and the singularity condition detJ=0, where *J* is the Jacobian of the deterministic drift. Near a nondegenerate fold, the model exhibits square-root equilibrium scaling and critical slowing down. Gillespie Monte Carlo simulations complement the analytical results by quantifying finite-horizon survival probability, extinction time, conditional bacterial burden, resistant fraction, and cumulative bacterial exposure. The resulting framework provides a mathematically consistent description of how bacterial growth, mutation, differential susceptibility, and limited treatment capacity jointly influence extinction and long-lived persistence.

## 1. Introduction

Antimicrobial resistance (AMR) is one of the major challenges facing modern infectious-disease treatment [1]. The effectiveness of antibiotic therapy depends not only on the intrinsic susceptibility of bacterial strains but also on the population dynamics of sensitive and resistant cells during treatment. Mathematical models of antimicrobial chemotherapy have therefore been used to investigate the emergence of resistant bacteria, the consequences of treatment pressure, and the conditions under which resistant populations can persist [2,3,4].

Resistance can arise through mutation and other genetic mechanisms, while antibiotic exposure creates differential selection between sensitive and resistant cells cells [5,6]. The population-level consequences of these mechanisms may be highly nonlinear. For example, a resistant subpopulation that is initially very small may remain negligible under sufficiently effective treatment, whereas a modest change in treatment intensity or bacterial growth conditions may allow resistant cells to increase and become the dominant population [7]. Deterministic models based on ordinary differential equations are useful for describing average population trajectories and equilibrium behavior. Still, they do not directly represent the random timing of individual birth, death, and mutation events. Stochastic population models are therefore particularly relevant when extinction, rare resistance emergence, and fluctuations in bacterial burden are important features of the system [8,9,10,11,12,13].

Previous stochastic studies have considered bacterial population growth, resistance emergence, and treatment-induced extinction using birth–death processes, stochastic differential equations, and related population models. For example, Lipsitch and Levin [4] developed mathematical models of population dynamics for antibiotic-sensitive and antibiotic-resistant bacteria during antimicrobial treatment, paying particular attention to resistance emergence and treatment failure. More recently, stochastic population models have been used to study the persistence and extinction of bacterial populations under antimicrobial pressure, demonstrating that stochastic fluctuations can substantially affect long-term population behavior [14]. These studies emphasize that persistence and extinction should be treated as stochastic population outcomes rather than inferred solely from deterministic average behavior [15,16].

An important mathematical consequence of modeling bacterial populations as a finite-state continuous-time Markov process is the presence of an absorbing extinction state. This feature makes stochastic extinction and long-lived non-extinct behavior distinct from the positive equilibria of the associated deterministic approximation. The subsequent sections develop the detailed stochastic and deterministic analyses. The model incorporates density-dependent bacterial growth, sensitive-to-resistant mutation, differential treatment susceptibility, and finite treatment capacity. These mechanisms provide a framework for studying how treatment capacity and mutation influence extinction, persistence, and equilibrium transitions. The principal objective of this work is to formulate the stochastic sensitive–resistant bacterial model, derive its exact first-moment dynamics, analyze the associated deterministic equilibria and their capacity-induced transitions, and assess the resulting stochastic behavior using Gillespie simulation. The main contributions of this study are summarized as follows:

1. We formulate a finite-state stochastic population model for antibiotic-sensitive and antibiotic-resistant bacteria that incorporates density-dependent growth, mutation, differential treatment susceptibility, carrying capacity, and capacity-limited bacterial removal.
2. We derive the exact first-moment equations and establish the role of mutation as a transfer mechanism between bacterial phenotypes rather than as an independent source of total population growth.
3. We rigorously distinguish the absorbing extinction state from long-lived non-extinct behavior and formulate the appropriate transient and quasi-stationary interpretation of the stochastic process.
4. We derive local extinction stability conditions and identify separate sensitive and resistant invasion boundaries based on the linearized deterministic dynamics.
5. We characterize positive equilibria in the different treatment-capacity regimes and develop analytical and numerical conditions for detecting capacity-induced critical transitions.
6. We establish the local square-root scaling and relaxation-time behavior associated with a nondegenerate fold transition, providing an alternative to classical heavy-traffic asymptotics.
7. We use Gillespie Monte Carlo simulations to quantify extinction, survival, bacterial burden, resistance composition, and cumulative bacterial exposure.
8. We demonstrate how treatment capacity, mutation, bacterial growth, and differential susceptibility jointly determine the transition between effective control and long-lived bacterial persistence.

The remainder of the paper is organized as follows. Section 2 introduces the stochastic population model and its transition rates. Section 3 analyzes extinction, first-moment dynamics, and local stability. Section 4 develops the deterministic equilibrium structure and threshold analysis under different treatment-capacity regimes. Section 5 investigates critical behavior near equilibrium transitions. Section 6 presents the Monte Carlo simulation methodology and numerical simulation results. Section 7 summarizes the principal findings, limitations, and directions for future work.

## 2. Stochastic Population Model Under Capacity-Limited Antibiotic Treatment

We formulate the dynamics of sensitive and antibiotic-resistant bacteria as a finite-state continuous-time Markov population process. The formulation explicitly distinguishes per capita bacterial growth and mutation rates from the capacity-limited removal mechanism induced by antibiotic treatment. This distinction is important because the bacterial populations are not supplied by an external arrival stream; rather, individuals reproduce and mutate within the population.

### 2.1. State Space and Model Assumptions

Let

$$N\left(t\right)=\left(N_{s}\left(t\right),N_{r}\left(t\right)\right),\;t\geq 0,$$

where $N_{s}\left(t\right)$ and $N_{r}\left(t\right)$ denote the numbers of antibiotic-sensitive and antibiotic-resistant bacteria at time *t*, respectively. The total bacterial population is

$$N\left(t\right)=N_{s}\left(t\right)+N_{r}\left(t\right).$$

We impose a finite carrying capacity K∈N and define the state space by

(1) $$S=\left\{\left(n_{s},n_{r}\right)\in Z_{\geq 0}^{2}:n_{s}+n_{r}\leq K\right\}.$$

The subset

$$A=\left\{\left(0,n_{r}\right):0\leq n_{r}\leq K\right\}\subset S$$

is absorbing. Indeed, once the sensitive population reaches zero, no transition mechanism regenerates sensitive bacteria. The state (0,0) is the unique extinction state within this absorbing set. Thus, the population cannot exceed the carrying capacity. The model incorporates five elementary biological events:

1. Reproduction of sensitive bacteria;
2. Reproduction of resistant bacteria;
3. Antibiotic-mediated removal of sensitive bacteria;
4. Antibiotic-mediated removal of resistant bacteria;
5. Mutation of sensitive bacteria into resistant bacteria.

The parameters $\lambda_{s}$ and $\lambda_{r}$ are the per capita proliferation rates of sensitive and resistant bacteria, respectively, while ν is the per capita mutation rate from the sensitive to the resistant population. The antibiotic killing rate for a sensitive bacterium is denoted by μ. Resistant bacteria are assumed to have reduced susceptibility, represented by the factor

0<ϵ<1.

Finally, c∈N denotes the effective treatment-capacity scale, representing the maximum number of bacterial killing events that can occur simultaneously under the treatment. Thus, *c* controls the saturation level of the treatment-removal mechanism. It is interpreted phenomenologically rather than as a literal number of molecular killing channels or as a direct antibiotic concentration. The corresponding maximum effective killing rates are μc for sensitive bacteria and ϵμc for resistant bacteria. In an experimental calibration, *c* can be related to measurable treatment characteristics through the effective maximal killing capacity μc using appropriate pharmacokinetic–pharmacodynamic or bacterial time–kill data.

### 2.2. State-Dependent Transition Rates

The proliferation of both bacterial types is regulated by the total population through a logistic factor. For a state $(n_{s},n_{r})\in S$, the sensitive-bacterial reproduction rate is

(2) $$b_{s}\left(n_{s},n_{r}\right)=\lambda_{s}n_{s}\left(1-\frac{n_{s}+n_{r}}{K}\right),$$

whereas the resistant-bacterial reproduction rate is

(3) $$b_{r}\left(n_{s},n_{r}\right)=\lambda_{r}n_{r}\left(1-\frac{n_{s}+n_{r}}{K}\right).$$

The logistic factor ensures that reproduction vanishes when the carrying capacity is reached. In particular,

$$b_{s}\left(n_{s},n_{r}\right)=b_{r}\left(n_{s},n_{r}\right)=0\;\mathrm{whenever}\;n_{s}+n_{r}=K.$$

Antibiotic-mediated removal is capacity-limited. The sensitive-bacterial death rate is

(4) $$d_{s}\left(n_{s},n_{r}\right)=\mu \min\left(n_{s},c\right),$$

whereas the resistant-bacterial death rate is

(5) $$d_{r}\left(n_{s},n_{r}\right)=\epsilon \mu \min\left(n_{r},c\right).$$

Consequently, when the number of bacteria of a given type exceeds *c*, the corresponding total removal rate is capped. In particular,

$$d_{s}\left(n_{s},n_{r}\right)\leq \mu c,\;d_{r}\left(n_{s},n_{r}\right)\leq \epsilon \mu c.$$

This saturation is a structural feature of the model and will be explicitly accounted for in the subsequent stability and equilibrium analysis.

Mutation converts one sensitive bacterium into one resistant bacterium at the state-dependent rate

(6) $$m\left(n_{s},n_{r}\right)=\nu n_{s}.$$

**Figure 1:**
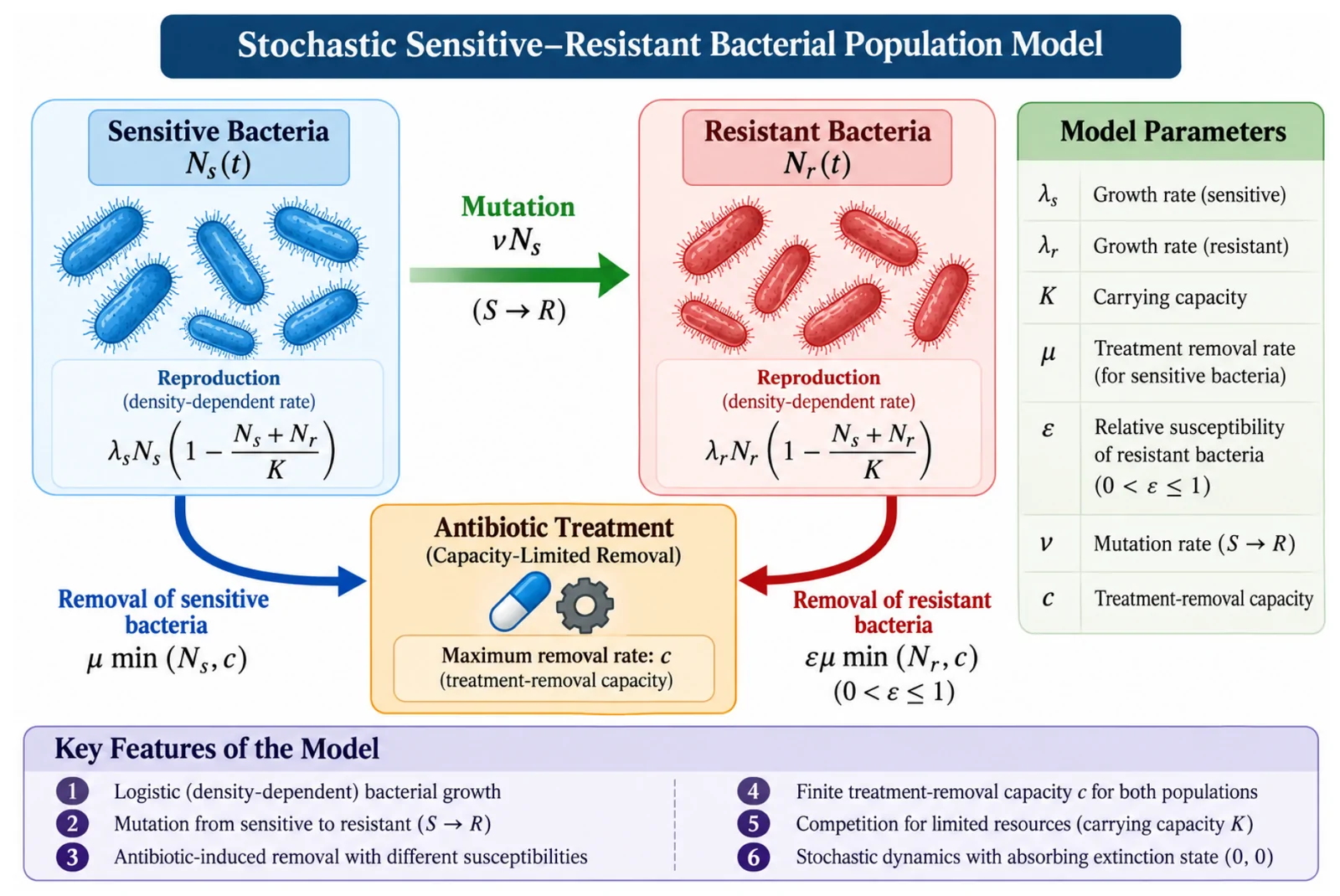
Schematic representation of the stochastic sensitive–resistant bacterial population model. Sensitive and resistant bacteria reproduce with density-dependent rates, sensitive bacteria mutate into the resistant class, and antibiotic treatment removes both populations with different susceptibilities under a finite treatment-removal capacity *c*.

Importantly, mutation does not change the total number of bacteria because

$$\left(n_{s},n_{r}\right)⟶\left(n_{s}-1,n_{r}+1\right)$$

leaves $n_{s}+n_{r}$ unchanged. A schematic overview of these mechanisms is shown in Figure 1.

### 2.3. Continuous-Time Markov Chain Representation

The process {N(t),t≥0} is a continuous-time Markov chain with the following nonzero transitions from a state $\left(n_{s},n_{r}\right)$:

(7) $$\begin{array}{cc} (n_{s},n_{r})⟶(n_{s}+1,n_{r}),\; & q_{\left(n_{s},n_{r}\right),\left(n_{s}+1,n_{r}\right)}=b_{s}\left(n_{s},n_{r}\right), \end{array}$$

(8) $$\begin{array}{cc} (n_{s},n_{r})⟶(n_{s},n_{r}+1),\; & q_{\left(n_{s},n_{r}\right),\left(n_{s},n_{r}+1\right)}=b_{r}\left(n_{s},n_{r}\right), \end{array}$$

(9) $$\begin{array}{cc} (n_{s},n_{r})⟶(n_{s}-1,n_{r}),\; & q_{\left(n_{s},n_{r}\right),\left(n_{s}-1,n_{r}\right)}=d_{s}\left(n_{s},n_{r}\right), \end{array}$$

(10) $$\begin{array}{cc} (n_{s},n_{r})⟶(n_{s},n_{r}-1),\; & q_{\left(n_{s},n_{r}\right),\left(n_{s},n_{r}-1\right)}=d_{r}\left(n_{s},n_{r}\right), \end{array}$$

(11) $$\begin{array}{cc} (n_{s},n_{r})⟶(n_{s}-1,n_{r}+1),\; & q_{\left(n_{s},n_{r}\right),\left(n_{s}-1,n_{r}+1\right)}=m(n_{s},n_{r}). \end{array}$$

Transitions that would leave the state space S are assigned rate zero. In particular, sensitive-bacterial reproduction and resistant-bacterial reproduction are automatically suppressed at the carrying-capacity boundary, while death and mutation transitions are possible only when the corresponding population is positive.

The total rate of leaving state $\left(n_{s},n_{r}\right)$ is, therefore,

(12) $$q\left(n_{s},n_{r}\right)=b_{s}\left(n_{s},n_{r}\right)+b_{r}\left(n_{s},n_{r}\right)+d_{s}\left(n_{s},n_{r}\right)+d_{r}\left(n_{s},n_{r}\right)+m\left(n_{s},n_{r}\right).$$

The diagonal elements of the generator are

(13) $$q_{\left(n_{s},n_{r}\right),\left(n_{s},n_{r}\right)}=-q\left(n_{s},n_{r}\right),$$

and all other off-diagonal entries are zero.

### 2.4. Infinitesimal Generator

Let

f:S→R

be any bounded function. The infinitesimal generator L of the continuous-time Markov chain is

(14) $$\begin{array}{cc} Lf(n_{s},n_{r})= & b_{s}\left(n_{s},n_{r}\right)\left[f\left(n_{s}+1,n_{r}\right)-f\left(n_{s},n_{r}\right)\right]\\ \; & +b_{r}\left(n_{s},n_{r}\right)\left[f\left(n_{s},n_{r}+1\right)-f\left(n_{s},n_{r}\right)\right]\\ \; & +d_{s}\left(n_{s},n_{r}\right)\left[f\left(n_{s}-1,n_{r}\right)-f\left(n_{s},n_{r}\right)\right]\\ \; & +d_{r}\left(n_{s},n_{r}\right)\left[f\left(n_{s},n_{r}-1\right)-f\left(n_{s},n_{r}\right)\right]\\ \; & +m\left(n_{s},n_{r}\right)\left[f\left(n_{s}-1,n_{r}+1\right)-f\left(n_{s},n_{r}\right)\right]. \end{array}$$

Equivalently, after substituting the transition rates,

(15) $$\begin{array}{cc} Lf(n_{s},n_{r})= & \lambda_{s}n_{s}\left(1-\frac{n_{s}+n_{r}}{K}\right)\left[f\left(n_{s}+1,n_{r}\right)-f\left(n_{s},n_{r}\right)\right]\\ \; & +\lambda_{r}n_{r}\left(1-\frac{n_{s}+n_{r}}{K}\right)\left[f\left(n_{s},n_{r}+1\right)-f\left(n_{s},n_{r}\right)\right]\\ \; & +\mu \min\left(n_{s},c\right)\left[f\left(n_{s}-1,n_{r}\right)-f\left(n_{s},n_{r}\right)\right]\\ \; & +\epsilon \mu \min\left(n_{r},c\right)\left[f\left(n_{s},n_{r}-1\right)-f\left(n_{s},n_{r}\right)\right]\\ \; & +\nu n_{s}\left[f\left(n_{s}-1,n_{r}+1\right)-f\left(n_{s},n_{r}\right)\right]. \end{array}$$

This generator completely specifies the stochastic population dynamics and will be used as the starting point for the subsequent analysis. In particular, no external arrival process is assumed: all population increases arise from endogenous bacterial reproduction, while mutation transfers individuals between the two bacterial types.

### 2.5. Forward Master Equation and Extinction State

Define the transient-state probability

(16) $$p_{n_{s},n_{r}}\left(t\right)=\mathrm{Pr}\left\{N_{s}\left(t\right)=n_{s},\;N_{r}\left(t\right)=n_{r}\right\}.$$

The probability vector

$$p\left(t\right)=\left\{p_{n_{s},n_{r}}\left(t\right):\left(n_{s},n_{r}\right)\in S\right\}$$

satisfies the forward Kolmogorov equation

(17) $$\frac{d}{dt}p\left(t\right)=p\left(t\right)Q,$$

where $Q={\left(q_{x,y}\right)}_{x,y\in S}$ is the generator matrix defined by (7)–(13).

The state

0=(0,0)

is absorbing. Indeed, when $n_{s}=n_{r}=0$,

$$b_{s}\left(0,0\right)=b_{r}\left(0,0\right)=d_{s}\left(0,0\right)=d_{r}\left(0,0\right)=m\left(0,0\right)=0.$$

Consequently,

(18) $$q_{\left(0,0),(0,0\right)}=0$$

and

Pr{N(t+h)=(0,0)∣N(t)=(0,0)}=1

for every h≥0.

This absorbing extinction state is an intrinsic component of the finite population model. Therefore, the long-term stochastic behavior cannot be characterized solely by an ordinary stationary distribution without first accounting for extinction. The distinction between extinction, persistence, and conditional long-term behavior is developed in Section 3.

## 3. Extinction, Persistence, and Stability Analysis

The finite-state structure of the stochastic population model has an important consequence for its long-term behavior. In particular, the extinction state is absorbing, and therefore the ordinary stationary distribution of the full process cannot be interpreted as a nontrivial persistent bacterial equilibrium. Accordingly, this section distinguishes three related notions: extinction of the bacterial population, local growth and decay near the extinction state, and conditional behavior before extinction. This distinction is essential for interpreting long-lived non-extinct dynamics in a finite stochastic population model.

### 3.1. Exact First-Moment Dynamics

Let

$$M_{s}\left(t\right)=E\left[N_{s}\left(t\right)\right],\;M_{r}\left(t\right)=E\left[N_{r}\left(t\right)\right]$$

denote the expected numbers of sensitive and resistant bacteria, respectively. Applying the generator in (15) to the coordinate functions

$$f_{s}\left(n_{s},n_{r}\right)=n_{s},\;f_{r}\left(n_{s},n_{r}\right)=n_{r},$$

gives the exact first-moment equations

(19) $$\begin{array}{cc} \frac{dM_{s}\left(t\right)}{dt}= & \lambda_{s}E\left[N_{s}\left(t\right)\left(1-\frac{N_{s}\left(t\right)+N_{r}\left(t\right)}{K}\right)\right]\\ \; & -\mu E\left[\min\{N_{s}\left(t\right),c\}\right]-\nu M_{s}\left(t\right), \end{array}$$

and

(20) $$\begin{array}{cc} \frac{dM_{r}\left(t\right)}{dt}= & \lambda_{r}E\left[N_{r}\left(t\right)\left(1-\frac{N_{s}\left(t\right)+N_{r}\left(t\right)}{K}\right)\right]\\ \; & -\epsilon \mu E\left[\min\{N_{r}\left(t\right),c\}\right]+\nu M_{s}\left(t\right). \end{array}$$

These equations are exact consequences of the Markov generator. In particular, they retain both the density-dependent proliferation terms and the capacity-limited antibiotic removal terms. The equations are not closed in terms of $\left(M_{s},M_{r}\right)$ alone because

$$E\left[N_{s}\left(1-\frac{N_{s}+N_{r}}{K}\right)\right]\neq M_{s}\left(1-\frac{M_{s}+M_{r}}{K}\right)$$

in general, and because

$$E\left[\min\left\{N_{i},c\right\}\right]\neq \min\left\{E\left[N_{i}\right],c\right\}.$$

Consequently, no unqualified linear mean-field stationary equations are used to represent the exact stochastic process.

The mutation terms cancel when the two equations are added, reflecting the fact that mutation changes bacterial type but does not change the total number of bacteria. Thus, for

$$M\left(t\right)=M_{s}\left(t\right)+M_{r}\left(t\right),$$

we obtain

(21) $$\begin{array}{cc} \frac{dM(t)}{dt}= & \lambda_{s}E\left[N_{s}\left(t\right)\left(1-\frac{N_{s}\left(t\right)+N_{r}\left(t\right)}{K}\right)\right]\\ \; & +\lambda_{r}E\left[N_{r}\left(t\right)\left(1-\frac{N_{s}\left(t\right)+N_{r}\left(t\right)}{K}\right)\right]\\ \; & -\mu E\left[\min\{N_{s}\left(t\right),c\}\right]-\epsilon \mu E\left[\min\{N_{r}\left(t\right),c\}\right]. \end{array}$$

Equation (21) emphasizes that the total population balance is determined by endogenous bacterial reproduction and capacity-limited treatment. Mutation affects the composition of the population but does not constitute an external source of additional bacteria.

### 3.2. Extinction as an Absorbing Event

Define the extinction time by

(22) $$\tau_{0}=\inf\left\{t\geq 0:N_{s}\left(t\right)+N_{r}\left(t\right)=0\right\}.$$

The state

0=(0,0)

is absorbing. Indeed, all transition rates in (2)–(6) vanish at (0,0). Hence,

(23) $$\mathrm{Pr}\left\{N(t)=(0,0)|N(0)=(0,0)\right\}=1\;\mathrm{for}\;\mathrm{all}\;t\geq 0.$$

Because the state space S is finite, extinction also has an important implication for the stationary behavior of the full Markov chain. From every nonzero state, there exists a finite sequence of positive-rate death transitions that reaches (0,0). Therefore, every state outside (0,0) can communicate, through a positive-probability path, with the absorbing state. Consequently, the only closed communicating class of the finite-state chain is

{(0,0)}.

It follows that the unique stationary distribution of the full finite-state process is the extinction distribution

(24) $$\pi^{\mathrm{ext}}\left(n_{s},n_{r}\right)=\left\{\begin{array}{cc} 1, & \left(n_{s},n_{r}\right)=\left(0,0\right),\\ 0, & \mathrm{otherwise}. \end{array}\right.$$

This observation is fundamental for the interpretation of the stochastic model. A nondegenerate stationary distribution with positive bacterial burden cannot be used as the stationary distribution of the original finite process. Long-lived bacterial populations observed before extinction must instead be described through transient, metastable, or quasi-stationary behavior.

### 3.3. Conditional Distribution Before Extinction

To characterize bacterial persistence prior to extinction, define the survival event

(25) $$S_{t}=\left\{\tau_{0}>t\right\}.$$

For states $\left(n_{s},n_{r}\right)\neq \left(0,0\right)$, the conditional distribution given survival is

(26) $${\tilde{\pi }}_{n_{s},n_{r}}\left(t\right)=\mathrm{Pr}\left\{N_{s}\left(t\right)=n_{s},\;N_{r}\left(t\right)=n_{r}|\tau_{0}>t\right\}.$$

**Figure 2:**
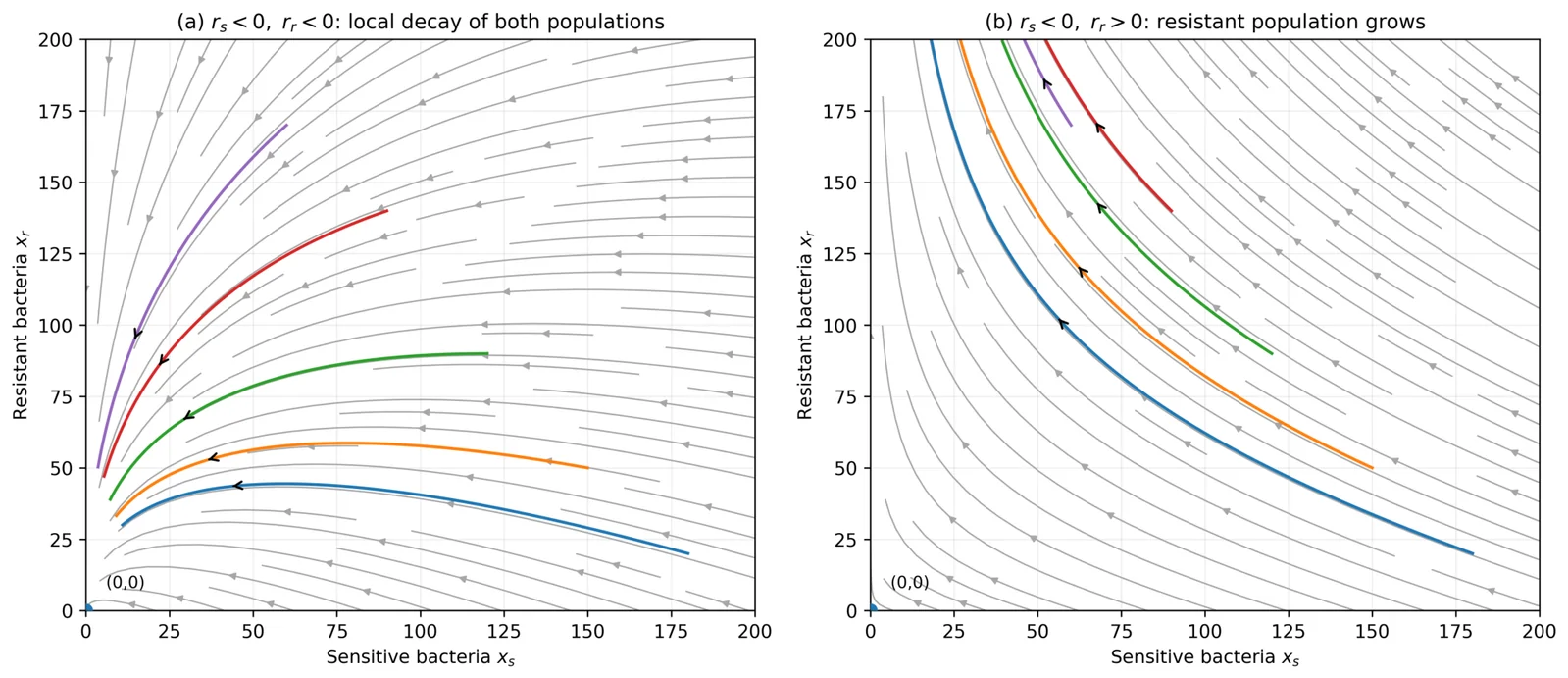
Phase-plane dynamics of the linearized sensitive–resistant bacterial model near the extinction state. Panel (**a**) corresponds to $r_{s}<0$ and $r_{r}<0$, for which trajectories decay toward the origin. Panel (**b**) corresponds to $r_{s}<0$ and $r_{r}>0$. In this case, $x_{s}\left(t\right)$ decreases monotonically whereas $x_{r}\left(t\right)$ increases monotonically, and trajectories move toward smaller $x_{s}$ and larger $x_{r}$, consistent with the analytical solution of the linearized system. The resistant component therefore grows exponentially as t→∞.

The corresponding local phase-plane behavior is illustrated in Figure 2.

### 3.4. Local Growth Rates near the Extinction State

Although the exact stochastic process is finite and absorbing, a local deterministic analysis near the extinction state provides a useful characterization of the initial growth or decay of small bacterial populations. Consider the drift obtained from the transition rates:

(27) $$\begin{array}{cc} F_{s}\left(x_{s},x_{r}\right)= & \lambda_{s}x_{s}\left(1-\frac{x_{s}+x_{r}}{K}\right)-\mu \min\left\{x_{s},c\right\}-\nu x_{s}, \end{array}$$

(28) $$\begin{array}{cc} F_{r}\left(x_{s},x_{r}\right)= & \lambda_{r}x_{r}\left(1-\frac{x_{s}+x_{r}}{K}\right)-\epsilon \mu \min\left\{x_{r},c\right\}+\nu x_{s}. \end{array}$$

For sufficiently small populations,

$$0\leq x_{s}<c,\;0\leq x_{r}<c,$$

and hence

$$\min\left\{x_{s},c\right\}=x_{s},\;\min\left\{x_{r},c\right\}=x_{r}.$$

Moreover, the logistic factors approach unity as $\left(x_{s},x_{r}\right)\to \left(0,0\right)$. The linearization at the extinction state is therefore

(29) $$\frac{d}{dt}\left(\begin{array}{c} x_{s}\\ x_{r} \end{array}\right)=J_{0}\left(\begin{array}{c} x_{s}\\ x_{r} \end{array}\right)+o\left(∥(x_{s},x_{r})∥\right),$$

where

(30) $$J_{0}=\left(\begin{array}{cc} \lambda_{s}-\mu -\nu & 0\\ \nu & \lambda_{r}-\epsilon \mu \end{array}\right).$$

Since $J_{0}$ is triangular, its eigenvalues are

(31) $$r_{s}=\lambda_{s}-\mu -\nu ,\;r_{r}=\lambda_{r}-\epsilon \mu .$$

The signs of the two local growth rates determine the initial growth or decay of small sensitive and resistant populations near the origin. In particular,

(32) $$r_{s}<0\;⟺\;\lambda_{s}<\mu +\nu ,\;r_{r}<0\;⟺\;\lambda_{r}<\epsilon \mu .$$

These inequalities describe the local growth–decay behavior of the deterministic drift and are not extinction criteria for the finite stochastic process. Mutation enters the off-diagonal component of the Jacobian and therefore transfers individuals from the sensitive to the resistant population without changing the resistant local growth eigenvalue $r_{r}$.

### 3.5. Interpretation of the Local Growth Conditions

The conditions in (32) should be interpreted as local conditions near the extinction state rather than as a global stationarity criterion for the finite stochastic process.

Three regimes are particularly relevant.

Locally controlled regime.

If

$$\lambda_{s}<\mu +\nu ,\;\lambda_{r}<\epsilon \mu ,$$

both local growth rates are negative. Small bacterial populations tend to decay under the local deterministic drift, and extinction is locally stable.

Sensitive-growth regime.

If

$$\lambda_{s}>\mu +\nu ,$$

the sensitive population has positive local growth in the absence of density-dependent suppression. In this regime, extinction is locally unstable in the sensitive direction.

Resistant-growth regime.

If

$$\lambda_{r}>\epsilon \mu ,$$

the resistant population has positive local growth near extinction. Because resistant bacteria are less susceptible to treatment, this condition identifies a fundamental mechanism through which resistant populations can persist despite antibiotic pressure. These local conditions are not equivalent to the existence of a nontrivial stationary distribution. The latter does not occur for the full finite absorbing process. Instead, positive long-lived bacterial burdens arise from the interaction of reproduction, mutation, carrying-capacity regulation, and capacity-limited treatment, and are investigated through the deterministic equilibrium structure and conditional stochastic behavior developed in the next section.

### 3.6. Consequences for Threshold Analysis

The preceding analysis shows that the extinction behavior is governed locally by two distinct growth conditions. In particular, $\lambda_{s}$, $\lambda_{r}$, and ν have different biological roles: $\lambda_{s}$ and $\lambda_{r}$ determine bacterial proliferation, whereas ν transfers individuals from the sensitive population to the resistant population without changing the total number of bacteria.

Accordingly, treatment control and resistance persistence cannot in general be represented by a single scalar threshold. The relevant transition conditions must instead be derived from the deterministic drift, the admissible positive equilibria, and the stability properties of those equilibria. These issues are developed in Section 4.

## 4. Positive Equilibria, Treatment Capacity, and Threshold Analysis

The local analysis of Section 3 characterizes the behavior of the system close to the extinction state. However, when bacterial populations become sufficiently large, density-dependent growth and treatment saturation become important. The resulting nonlinear dynamics may therefore admit positive equilibrium states representing long-lived bacterial populations before eventual stochastic extinction.

### 4.1. Deterministic Fluid Approximation

Let

$$x_{s}\left(t\right)\approx E\left[N_{s}\left(t\right)\right],\;x_{r}\left(t\right)\approx E\left[N_{r}\left(t\right)\right]$$

denote the deterministic population variables associated with the sensitive and resistant bacterial populations.

The corresponding fluid approximation is

(33) $$\frac{dx_{s}}{dt}=\lambda_{s}x_{s}\left(1-\frac{x_{s}+x_{r}}{K}\right)-\mu \min\left(x_{s},c\right)-\nu x_{s},$$

(34) $$\frac{dx_{r}}{dt}=\lambda_{r}x_{r}\left(1-\frac{x_{s}+x_{r}}{K}\right)-\epsilon \mu \min\left(x_{r},c\right)+\nu x_{s}.$$

Define the deterministic drift

$$F\left(x_{s},x_{r}\right)=\left(\begin{array}{c} F_{s}\left(x_{s},x_{r}\right)\\ F_{r}\left(x_{s},x_{r}\right) \end{array}\right),$$

where $F_{s}$ and $F_{r}$ denote the right-hand sides of Equations (27) and (28), respectively.

A positive equilibrium

$$\left(x_{s}^{*},x_{r}^{*}\right)$$

satisfies

$$F_{s}\left(x_{s}^{*},x_{r}^{*}\right)=0,\;F_{r}\left(x_{s}^{*},x_{r}^{*}\right)=0,$$

It is convenient to introduce the equilibrium density factor

$$q^{*}=1-\frac{x_{s}^{*}+x_{r}^{*}}{K}.$$

The carrying-capacity formulation restricts the physically meaningful state space to

$$0<x_{s}+x_{r}\leq K.$$

Therefore, at a positive equilibrium,

$$0<x_{s}^{*}+x_{r}^{*}\leq K,$$

and hence

$$0\leq q^{*}<1.$$

The equilibrium equations can then be written compactly as

(35) $$x_{s}^{*}\left(\lambda_{s}q^{*}-\nu \right)=\mu \min(x_{s}^{*},c),$$

(36) $$\lambda_{r}q^{*}x_{r}^{*}+\nu x_{s}^{*}=\epsilon \mu \min(x_{r}^{*},c).$$

**Figure 3:**
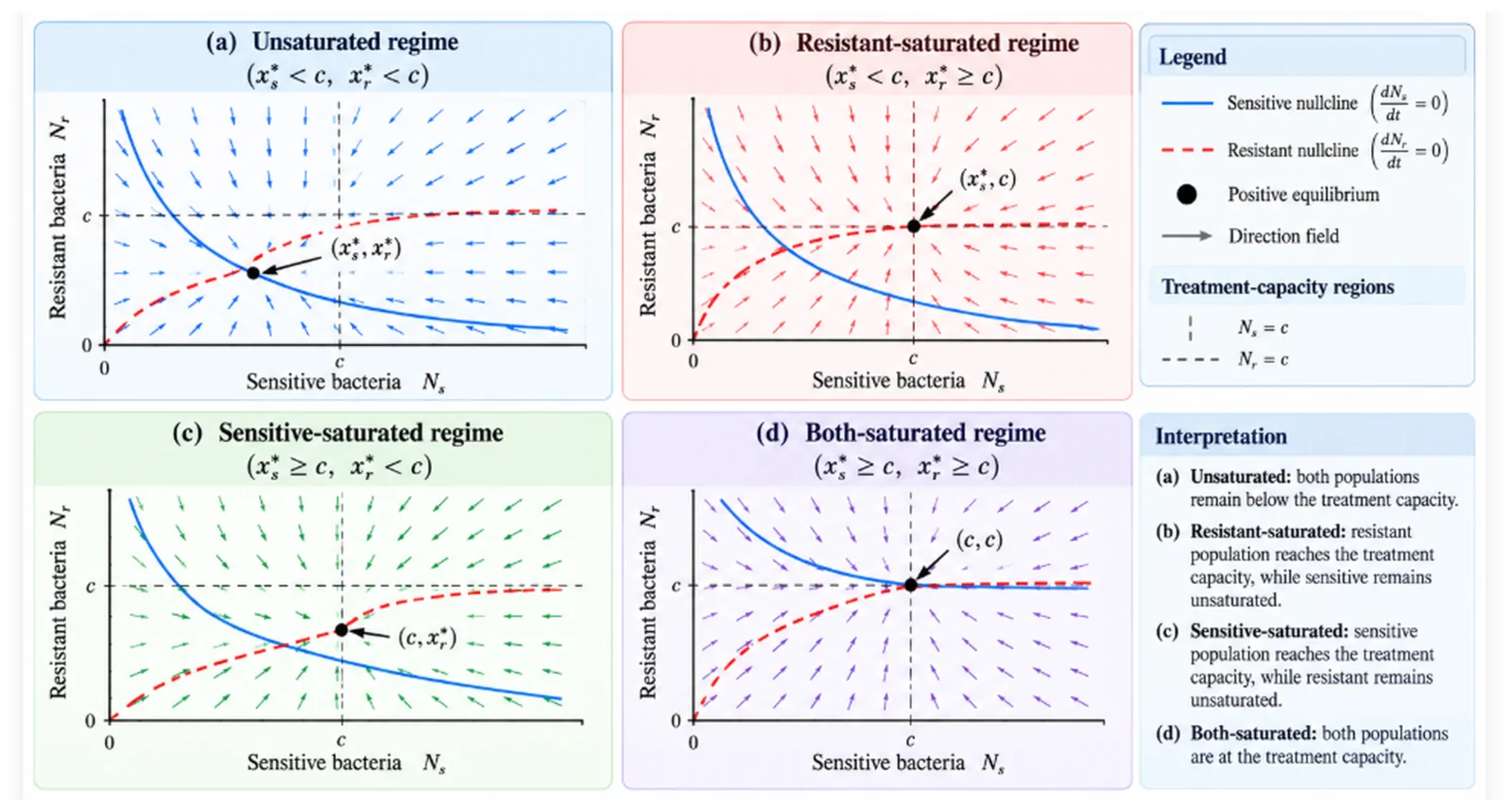
Positive equilibrium structure of the deterministic fluid model under different treatment-capacity regimes. The four panels correspond to the unsaturated, resistant-saturated, sensitive-saturated, and both-saturated regimes. The equilibrium location is determined by the intersection of the sensitive and resistant nullclines together with the corresponding capacity constraints. Blue and red dashed curves represent the sensitive and resistant nullclines, respectively. Gray arrows indicate the direction field, while colored curves represent solution trajectories from different initial conditions. Black markers indicate positive equilibrium points.

These equations provide the basis for the different treatment-capacity regimes considered below. The corresponding equilibrium regimes are illustrated in Figure 3.

### 4.2. Unsaturated Equilibrium Regime

First suppose that both populations remain below the treatment capacity:

$$x_{s}^{*}<c,\;x_{r}^{*}<c.$$

In this case,

$$\min\left(x_{s}^{*},c\right)=x_{s}^{*},\;\min\left(x_{r}^{*},c\right)=x_{r}^{*}.$$

The sensitive equilibrium equation becomes

$$x_{s}^{*}\left(\lambda_{s}q^{*}-\mu -\nu \right)=0.$$

For a positive sensitive equilibrium,

$$x_{s}^{*},$$

and therefore

(37) $$q^{*}=\frac{\mu +\nu }{\lambda_{s}}.$$

A positive equilibrium requires

$$0<q^{*}<1,$$

which implies

$$\lambda_{s}>\mu +\nu .$$

The resistant equilibrium equation becomes

$$\left(\epsilon \mu -\lambda_{r}q^{*}\right)x_{r}^{*}=\nu x_{s}^{*}.$$

Provided that

$$\epsilon \mu -\lambda_{r}q^{*}>0,$$

we obtain

(38) $$x_{r}^{*}=\frac{\nu x_{s}^{*}}{\epsilon \mu -\lambda_{r}q^{*}}.$$

Since

$$x_{s}^{*}+x_{r}^{*}=K\left(1-q^{*}\right),$$

substitution of (38) gives

(39) $$x_{s}^{*}=\frac{K(1-q^{*})}{1+\frac{\nu }{\epsilon \mu -\lambda_{r}q^{*}}},$$

and hence

(40) $$x_{r}^{*}=\frac{K(1-q^{*})\nu }{\epsilon \mu -\lambda_{r}q^{*}+\nu }.$$

### 4.3. Sensitive-Unsaturated and Resistant-Saturated Regime

Consider the next regime

$$x_{s}^{*}<c,\;x_{r}^{*}\geq c.$$

The sensitive equation remains

$$q^{*}=\frac{\mu +\nu }{\lambda_{s}}.$$

For the resistant population, treatment is saturated and therefore

$$\lambda_{r}q^{*}x_{r}^{*}+\nu x_{s}^{*}=\epsilon \mu c.$$

Using

$$x_{r}^{*}=K\left(1-q^{*}\right)-x_{s}^{*},$$

we obtain

$$\lambda_{r}q^{*}\left[K\left(1-q^{*}\right)-x_{s}^{*}\right]+\nu x_{s}^{*}=\epsilon \mu c.$$

Solving for $x_{s}^{*}$ gives

(41) $$x_{s}^{*}=\frac{\epsilon \mu c-\lambda_{r}q^{*}K\left(1-q^{*}\right)}{\nu -\lambda_{r}q^{*}}.$$

The resistant equilibrium is then

(42) $$x_{r}^{*}=K\left(1-q^{*}\right)-x_{s}^{*}.$$

The solution is admissible only when

$$x_{s}^{*}>0,\;x_{r}^{*}>0.$$

### 4.4. Sensitive-Saturated and Resistant-Unsaturated Regime

Now consider

$$x_{s}^{*}\geq c,\;x_{r}^{*}<c.$$

The sensitive population is treatment-saturated, so

$$\lambda_{s}q^{*}x_{s}^{*}-\mu c-\nu x_{s}^{*}=0.$$

Consequently,

(43) $$x_{s}^{*}=\frac{\mu c}{\lambda_{s}q^{*}-\nu }.$$

For the resistant population,

$$\lambda_{r}q^{*}x_{r}^{*}+\nu x_{s}^{*}=\epsilon \mu x_{r}^{*},$$

which gives

(44) $$x_{r}^{*}=\frac{\nu x_{s}^{*}}{\epsilon \mu -\lambda_{r}q^{*}}.$$

Since

$$x_{s}^{*}+x_{r}^{*}=K\left(1-q^{*}\right),$$

the unknown $q^{*}$ is determined by the scalar equation

(45) $$\frac{\mu c}{\lambda_{s}q^{*}-\nu }\left(1+\frac{\nu }{\epsilon \mu -\lambda_{r}q^{*}}\right)=K\left(1-q^{*}\right).$$

Equation (45) may have two algebraic roots in the interval

$$\frac{\nu }{\lambda_{s}}<q^{*}<\frac{\epsilon \mu }{\lambda_{r}}.$$

However, the existence of two algebraic roots does not imply the existence of two admissible equilibria in the same capacity regime. Each root must be checked against the regime conditions

$$x_{s}^{*}\geq c,\;x_{r}^{*}<c.$$

For the parameter set

$$\mu =1,\;\epsilon =0.1,\;c=5,\;K=1000,\;\lambda_{s}=1.8,\;\lambda_{r}=0.01,\;\nu =10^{-4},$$

Equation (45) has two roots,

$$q_{1}^{*}\approx 0.002844,\;q_{2}^{*}\approx 0.997211.$$

The first root gives

$$\left(x_{s}^{*},x_{r}^{*}\right)\approx \left(996.160,0.996\right),$$

which satisfies $x_{s}^{*}\geq c$ and $x_{r}^{*}<c$ and is therefore admissible for the sensitive-saturated/resistant-unsaturated regime. In contrast, the second root gives

$$\left(x_{s}^{*},x_{r}^{*}\right)\approx \left(2.786,0.003\right),$$

which violates $x_{s}^{*}\geq c$ and hence is not admissible in this regime. It belongs instead to the both-unsaturated capacity regime. Thus, the numerical calculation confirms that two algebraic roots may occur, while only one corresponds to an admissible equilibrium under the assumed capacity configuration.

### 4.5. Both Populations Saturated

Finally, suppose that

$$x_{s}^{*}\geq c,\;x_{r}^{*}\geq c.$$

Both removal mechanisms are then capacity-limited. The sensitive equilibrium equation gives

(46) $$x_{s}^{*}=\frac{\mu c}{\lambda_{s}q^{*}-\nu }.$$

The resistant equilibrium equation becomes

$$\lambda_{r}q^{*}x_{r}^{*}+\nu x_{s}^{*}=\epsilon \mu c,$$

and therefore

(47) $$x_{r}^{*}=\frac{\epsilon \mu c-\nu x_{s}^{*}}{\lambda_{r}q^{*}}.$$

Substitution into

$$x_{s}^{*}+x_{r}^{*}=K\left(1-q^{*}\right)$$

gives the scalar equilibrium equation

(48) $$\frac{\mu c}{\lambda_{s}q^{*}-\nu }+\frac{\epsilon \mu c-\frac{\nu \mu c}{\lambda_{s}q^{*}-\nu }}{\lambda_{r}q^{*}}=K\left(1-q^{*}\right).$$

For the representative parameter set

$$\mu =1,\;\epsilon =0.1,\;c=5,\;K=1000,\;\lambda_{s}=1.8,\;\lambda_{r}=0.01,\;\nu =10^{-4},$$

Equation (48) has three algebraic roots,

$$q_{1}^{*}\approx 0.000585,\;q_{2}^{*}\approx 0.055341,\;q_{3}^{*}\approx 0.944130.$$

Substitution into Equations (46) and (47) gives

$$\left(x_{s}^{*},x_{r}^{*}\right)\approx \left(5248.49,-4249.07\right),\;\left(50.24,894.41\right),\;\left(2.94,52.93\right),$$

respectively. Only the second root satisfies

$$x_{s}^{*}\geq c,\;x_{r}^{*}\geq c,$$

and is therefore admissible for the both-saturated regime. The other roots violate the assumed capacity conditions. Thus, as in the preceding regime, multiple algebraic roots do not imply multiple admissible equilibria.

### 4.6. Local Invasion Thresholds

The local stability analysis of Section 3 gives two natural invasion boundaries:

$$\lambda_{s}=\mu +\nu$$

and

$$\lambda_{r}=\epsilon \mu .$$

Equivalently, using the local invasion ratios

$$R_{s}=\frac{\lambda_{s}}{\mu +\nu },\;R_{r}=\frac{\lambda_{r}}{\epsilon \mu },$$

the corresponding boundaries are

$$R_{s}=1,\;R_{r}=1.$$

These quantities describe whether each bacterial type can increase when rare and the treatment-removal mechanism is unsaturated. They should not be interpreted as universal global thresholds for the full nonlinear model.

In particular, the condition

$$\lambda_{r}>\epsilon \mu$$

indicates local resistant growth near extinction, whereas a resistant population generated by mutation may also persist temporarily even when

$$\lambda_{r}<\epsilon \mu ,$$

because the mutation flux

$$\nu x_{s}$$

continuously supplies resistant bacteria while sensitive bacteria are present.

### 4.7. Capacity-Induced Persistence Threshold

The finite treatment capacity introduces an additional transition mechanism that is not captured by the local invasion ratios. Define the equilibrium system

$$F_{s}\left(x_{s},x_{r};\theta \right)=0,\;F_{r}\left(x_{s},x_{r};\theta \right)=0,$$

where θ denotes a parameter of interest, such as

$$\theta \in \{\lambda_{s},\lambda_{r},\nu ,\mu ,\epsilon ,c,K\}.$$

Let

$$J\left(x_{s},x_{r};\theta \right)=\left(\begin{array}{cc} \frac{\partial F_{s}}{\partial x_{s}} & \frac{\partial F_{s}}{\partial x_{r}}\\ \frac{\partial F_{r}}{\partial x_{s}} & \frac{\partial F_{r}}{\partial x_{r}} \end{array}\right)$$

denote the Jacobian matrix of the deterministic drift within a smooth capacity regime. A nondegenerate saddle-node (fold) transition is identified by

(49) $$F_{s}\left(x_{s}^{*},x_{r}^{*};\theta_{c}\right)=0,\;F_{r}\left(x_{s}^{*},x_{r}^{*};\theta_{c}\right)=0,$$

together with

(50) $$\det J(x_{s}^{*},x_{r}^{*};\theta_{c})=0.$$

At such a point, one eigenvalue of the Jacobian crosses zero. The condition (50) therefore provides a mathematical criterion for detecting a capacity-induced equilibrium transition. The critical point is determined by the nonlinear population balance and the singularity of the equilibrium Jacobian. Thus, the transition emerges from the interaction between bacterial growth, mutation, differential treatment susceptibility, carrying capacity, and treatment saturation, rather than from a single scalar threshold condition.

### 4.8. Numerical Determination of Critical Parameters

For a selected parameter θ, the critical value can be determined numerically through the following procedure.

1. Choose a parameter interval
  $$[\theta_{\min},\theta_{\max}].$$
2. For each value of θ, solve
  $$F_{s}\left(x_{s},x_{r};\theta \right)=0,\;F_{r}\left(x_{s},x_{r};\theta \right)=0.$$
3. Determine the capacity regime associated with each equilibrium and retain only solutions satisfying the corresponding inequalities.
4. Compute the Jacobian
  $$J(x_{s}^{*},x_{r}^{*};\theta )$$
  and its eigenvalues
  $$\zeta_{1}\left(\theta \right),\;\zeta_{2}\left(\theta \right).$$
5. Identify a candidate critical point when the dominant eigenvalue approaches zero:
  maxiRe{ζi(θ)}→0.
6. Confirm the transition by solving
  $$F_{s}=0,\;F_{r}=0,\;\det J=0$$
  simultaneously.
7. Finally, validate the predicted critical parameter using finite-horizon stochastic simulations based on the Gillespie algorithm.

This procedure separates the analytical identification of a nonlinear critical point from its stochastic validation.

### 4.9. Interpretation of the Threshold Structure

The model therefore contains several distinct threshold concepts.

First, the local extinction boundaries

$$\lambda_{s}=\mu +\nu \;\mathrm{and}\;\lambda_{r}=\epsilon \mu$$

describe the ability of bacterial populations to grow when rare. Second, positive-equilibrium transitions arise from the nonlinear interaction between logistic growth and finite treatment capacity. Third, stochastic treatment outcomes can be characterized using finite-horizon quantities such as

$$S\left(T\right)=P\left(\tau_{0}>T\right),$$

the conditional bacterial burden

$$E[N_{s}\left(T\right)+N_{r}\left(T\right)|\tau_{0}>T],$$

and the extinction time $\tau_{0}$.

These three notions should not be collapsed into a single critical mutation rate. In particular, the mutation parameter ν does not generally define a universal threshold of the form

$$\nu_{c}=c\epsilon \mu -\lambda_{s}.$$

Instead, increasing ν modifies the sensitive population through the loss term $-\nu x_{s}$ and simultaneously supplies resistant bacteria through the transfer term $+\nu x_{s}$. Its overall effect therefore depends on the nonlinear equilibrium structure and on the treatment-capacity regime.

The threshold analysis developed here consequently provides a parameter-dependent description of treatment control and resistance persistence based on the nonlinear equilibrium structure of the population process.

## 5. Critical Behavior near Capacity and Equilibrium Transitions

The finite-state stochastic model considered in this study has an absorbing extinction state. Consequently, the critical behavior of the system should not be interpreted as a divergence of a stationary population measure. Instead, the relevant transition is associated with the existence, stability, and disappearance of positive equilibria of the deterministic approximation. These equilibria describe long-lived population states around which the stochastic process may remain for extended periods before eventual extinction.

The analysis in this section therefore focuses on capacity-induced transitions and their relation to the local stability of the extinction state. In particular, no scalar traffic intensity is introduced, and no ordinary stationary-load divergence is assumed.

### 5.1. Equilibrium Transitions and Critical Parameters

Let

$$x\left(t\right)=\left(\begin{array}{c} x_{s}\left(t\right)\\ x_{r}\left(t\right) \end{array}\right),$$

where $x_{s}\left(t\right)$ and $x_{r}\left(t\right)$ denote the deterministic approximations to the numbers of sensitive and resistant bacteria, respectively. From $F_{s}$ and $F_{r}$ are the deterministic drift components defined by Equations (27) and (28). An equilibrium $x^{*}=\left(x_{s}^{*},x_{r}^{*}\right)$ satisfies

(51) $$F_{s}\left(x_{s}^{*},x_{r}^{*}\right)=0,\;F_{r}\left(x_{s}^{*},x_{r}^{*}\right)=0.$$

The equilibrium structure depends on whether each bacterial population is below or above the treatment capacity *c*. Hence, the system is piecewise smooth, with different equilibrium equations in the four regimes

$$\left(x_{s}^{*},x_{r}^{*}\right)\in \left\{\left(x_{s}<c,x_{r}<c\right),\;\left(x_{s}<c,x_{r}\geq c\right),\;\left(x_{s}\geq c,x_{r}<c\right),\;\left(x_{s}\geq c,x_{r}\geq c\right)\right\}.$$

This piecewise structure is important because the treatment term changes from a population-dependent removal rate to a capacity-limited removal rate. Therefore, the critical behavior cannot in general be characterized by a single expression involving only $\lambda_{s}$, ν, μ, and ϵ.

### 5.2. Local Stability of Positive Equilibria

Suppose that $x^{*}$ lies strictly inside one of the four smooth capacity regimes. The local behavior around this equilibrium is determined by the Jacobian matrix

(52) $$J\left(x^{*}\right)={\left[\begin{array}{cc} \frac{\partial F_{s}}{\partial x_{s}}\; & \frac{\partial F_{s}}{\partial x_{r}}\\ \frac{\partial F_{r}}{\partial x_{s}}\; & \frac{\partial F_{r}}{\partial x_{r}} \end{array}\right]}_{x=x^{*}}.$$

For a non-saturated population, the derivative of the corresponding treatment term is μ for the sensitive population and ϵμ for the resistant population. For a saturated population, the corresponding derivative of the treatment term is zero because the removal rate has reached its capacity. For example, in the regime

$$x_{s}^{*}<c,\;x_{r}^{*}<c,$$

the Jacobian is

(53) $$J\left(x^{*}\right)=\left(\begin{array}{cc} \lambda_{s}\left(1-\frac{2x_{s}^{*}+x_{r}^{*}}{K}\right)-\mu -\nu \; & -\frac{\lambda_{s}x_{s}^{*}}{K}\\ -\frac{\lambda_{r}x_{r}^{*}}{K}+\nu \; & \lambda_{r}\left(1-\frac{x_{s}^{*}+2x_{r}^{*}}{K}\right)-\epsilon \mu \end{array}\right).$$

The equilibrium is locally asymptotically stable when both eigenvalues of $J(x^{*})$ have negative real parts. For a two-dimensional system, this is equivalently expressed as

(54) $$\mathrm{tr}\left(J(x^{*})\right)<0,\;\det\left(J(x^{*})\right)>0.$$

These conditions provide a direct stability test for each positive equilibrium obtained from the piecewise equilibrium equations.

### 5.3. Capacity-Induced Critical Points

A critical transition may occur when a stable positive equilibrium collides with an unstable equilibrium and disappears. Such a transition is naturally described by a saddle-node, or fold, condition. Let θ denote a parameter whose variation is used to study the transition. Depending on the application, θ may be the mutation rate ν, the treatment capacity *c*, the sensitive growth rate $\lambda_{s}$, the resistant growth rate $\lambda_{r}$, or another model parameter. A candidate critical point $\left(x_{c},\theta_{c}\right)$ satisfies

(55) $$F(x_{c};\theta_{c})=0,$$

together with

(56) $$\det J(x_{c};\theta_{c})=0.$$

Thus, the critical parameter is determined jointly from

(57) $$\begin{array}{c} F_{s}\left(x_{s}^{*},x_{r}^{*};\theta \right)=0,\;F_{r}\left(x_{s}^{*},x_{r}^{*};\theta \right)=0,\;\det J\left(x_{s}^{*},x_{r}^{*};\theta \right)=0. \end{array}$$

For the critical treatment-capacity value identified in Section 5, additional Gillespie simulations were performed at

c∈{129.0,129.5723,130.0},

using

$$\mu =1,\;\epsilon =0.1,\;K=10^{4},\;\lambda_{s}=0.75,\;\lambda_{r}=0.035,\;\nu =0.0015,$$

with

$$N_{s}\left(0\right)=6309,\;N_{r}\left(0\right)=3397,\;T=100,\;M=10^{4}.$$

The finite-horizon survival probability, conditional bacterial burden, and conditional resistant fraction were recorded for each value of *c*. The corresponding Monte Carlo estimates and uncertainty intervals are reported in Table 1.

This formulation explicitly incorporates population growth, mutation, differential susceptibility, carrying capacity, and treatment capacity. Consequently, it avoids reducing the critical behavior to a single traffic-intensity condition. For a nondegenerate fold, the Jacobian has one simple zero eigenvalue while the remaining eigenvalue is nonzero. Additional transversality and nondegeneracy conditions determine whether the bifurcation is a genuine saddle-node transition. In practical applications, these conditions can be verified numerically from the Jacobian and the parameter-dependent drift.

### 5.4. Local Scaling near a Nondegenerate Fold

The local behavior near a nondegenerate fold can be described by a one-dimensional normal form. Let *u* denote the coordinate along the critical eigenvector and let $\delta =\theta -\theta_{c}$. Under the standard nondegeneracy conditions, the dynamics locally reduce to

(58) $$\frac{du}{dt}=a\delta +bu^{2}+o\left(\left|\delta \right|+u^{2}\right),$$

where a≠0 and b≠0.

The nearby equilibria therefore satisfy

(59) $$a\delta +bu^{2}\approx 0,$$

which gives

(60) $$u^{*}\approx \pm \sqrt{-\frac{a}{b}\delta }.$$

Hence, when a positive equilibrium disappears through a nondegenerate fold, the distance of the equilibrium from the critical state scales as

(61) $$\begin{array}{c} |u^{*}-u_{c}|\propto |\theta -\theta_{c}{|}^{1/2}. \end{array}$$

This square-root scaling is a local property of the deterministic equilibrium transition and follows directly from the nondegenerate fold structure. It characterizes the response of the equilibrium branch as the control parameter approaches its critical value.

### 5.5. Critical Slowing Down of the Deterministic Dynamics

The same local normal form provides information about the relaxation rate. Linearization around a stable equilibrium gives

(62) $$\frac{d}{dt}\delta x=J\left(x^{*}\right)\delta x.$$

Let $\Lambda_{\max}$ denote the eigenvalue with the largest real part. The local relaxation time is approximately

(63) τrel=−1Re(Λmax),

provided Re(Λmax)<0.

As a stable equilibrium approaches a nondegenerate fold, the leading eigenvalue approaches zero. Using the normal form above gives

(64) $$|\Lambda_{\max}|\propto |\theta -\theta_{c}{|}^{1/2},$$

and therefore

(65) $$\begin{array}{c} \tau_{\mathrm{rel}}\propto {\left|\theta -\theta_{c}\right|}^{-1/2}. \end{array}$$

**Figure 4:**
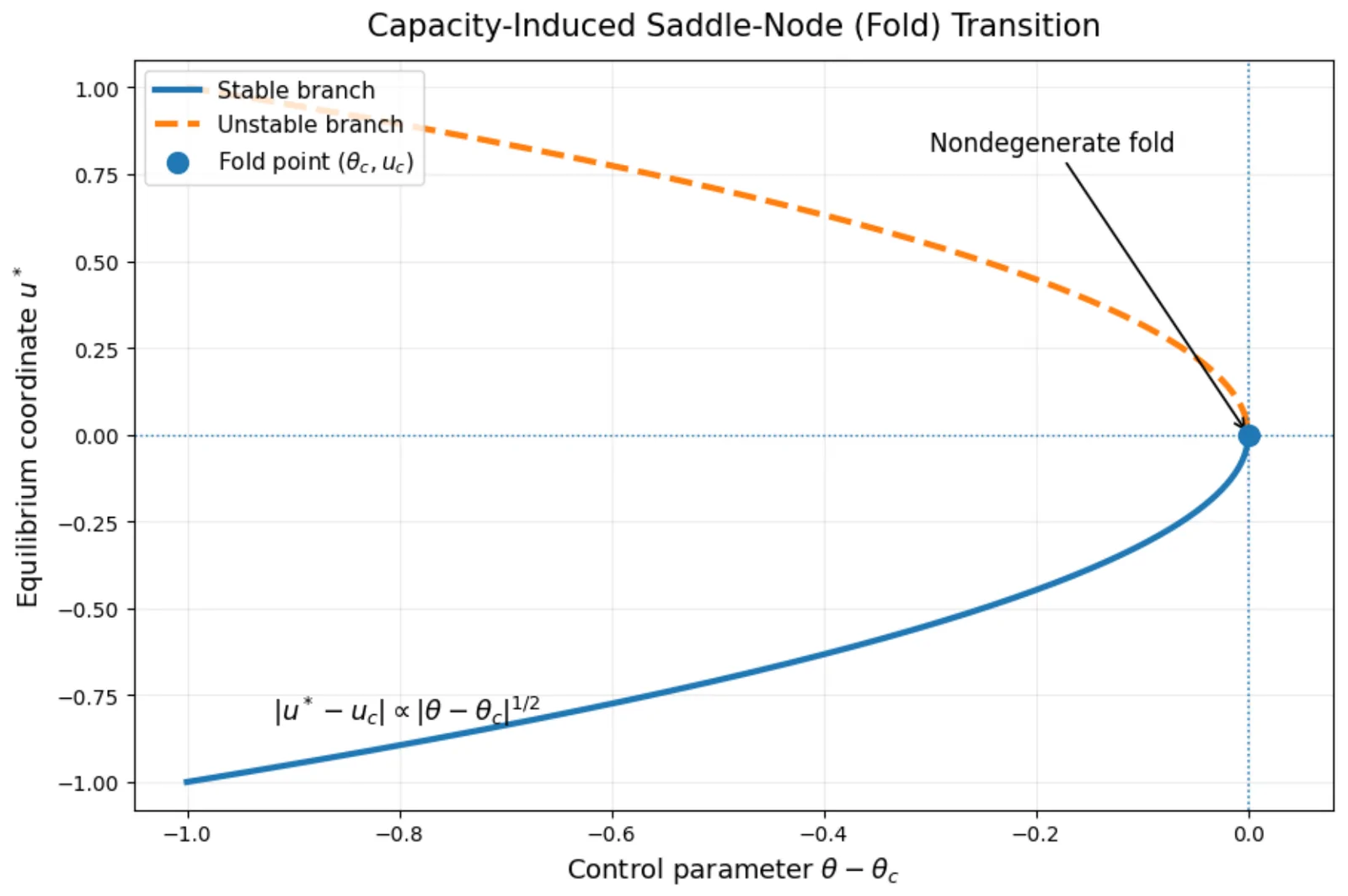
Schematic representation of a nondegenerate saddle-node (fold) transition in the deterministic approximation. The stable and unstable positive-equilibrium branches approach each other as the control parameter θ approaches the critical value $\theta_{c}$. At the fold, the leading Jacobian eigenvalue reaches zero and the positive equilibrium disappears. The local square-root scaling $|u^{*}-u_{c}|\propto |\theta -\theta_{c}{|}^{1/2}$ follows from the normal form derived in Section 5. This figure is a mathematical schematic and is not based on a numerical simulation.

Thus, the model may exhibit critical slowing down as a positive equilibrium approaches a capacity-induced fold. This result follows directly from the local deterministic dynamics and the nondegenerate fold structure. The resulting fold structure is illustrated schematically in Figure 4.

### 5.6. Relation Between Local Extinction and Positive-Equilibrium Transitions

Two distinct mechanisms must be distinguished. First, the extinction state is locally stable when

(66) $$\lambda_{s}<\mu +\nu ,\;\lambda_{r}<\epsilon \mu .$$

Second, a positive equilibrium may exist and remain locally stable because the nonlinear logistic and capacity-limited terms modify the dynamics away from the origin. Consequently, local stability of the extinction state does not by itself rule out the existence of a positive attracting state.

This distinction is particularly important when treatment capacity is small relative to the bacterial population. Once a population exceeds the effective treatment capacity, the corresponding removal rate becomes approximately constant rather than increasing proportionally with population size. Logistic growth can then generate a nonlinear balance that permits positive equilibria even when the extinction state remains locally stable.

Therefore, the model can potentially exhibit coexistence of a locally stable extinction state and a locally stable positive equilibrium, separated by an unstable equilibrium. Such a configuration provides a deterministic mechanism for bistability and sharp transitions in treatment outcome.

### 5.7. Mutation as a Control Parameter

The mutation rate ν has two distinct mathematical effects. In the sensitive equation, it contributes to the loss of sensitive bacteria through the term $-\nu x_{s}$, while in the resistant equation it contributes the transfer term $+\nu x_{s}$. Hence, mutation does not create bacteria; it redistributes individuals between the two phenotypic classes. At the extinction state, increasing ν changes the sensitive local growth rate according to

(67) $$r_{s}=\lambda_{s}-\mu -\nu ,$$

while the resistant local growth rate remains

(68) $$r_{r}=\lambda_{r}-\epsilon \mu .$$

Therefore, the local resistant invasion boundary is not obtained by solving for ν alone. The effect of mutation on long-term treatment outcome is instead determined by the nonlinear positive-equilibrium structure and by the resulting resistant population. For this reason, a universal mutation threshold of the form

$$\nu_{c}=c\epsilon \mu -\lambda_{s}$$

is not used in the present formulation. A critical mutation value, when it exists, is obtained from the equilibrium and fold conditions

(69) $$F_{s}=0,\;F_{r}=0,\;\det\left(J\right)=0,$$

with all regime constraints satisfied.

### 5.8. Numerical Identification of a Critical Fold Transition

To demonstrate that the fold condition derived above can occur for biologically admissible parameter values, we numerically solve the system

(70) $$F_{s}\left(x_{s},x_{r};c\right)=0,\;F_{r}\left(x_{s},x_{r};c\right)=0,\;\det J\left(x_{s},x_{r};c\right)=0.$$

As a representative parameter set, we consider

(71) $$\mu =1,\;\epsilon =0.1,\;K=10^{4},\;\lambda_{s}=0.75,\;\lambda_{r}=0.035,\;\nu =0.0015.$$

The numerical solution of the above system gives the critical treatment capacity

(72) $$c_{c}=129.5723,$$

with the corresponding critical equilibrium

(73) $$\left(x_{s}^{c},x_{r}^{c}\right)=\left(6308.69,\;3397.46\right).$$

At this point, the eigenvalues of the Jacobian are

(74) $$\Lambda_{1}=-0.46348,\;\Lambda_{2}\approx 0,$$

confirming the singularity of the equilibrium Jacobian at the critical point. Numerical continuation with respect to *c* confirms the fold structure. For $c<c_{c}$, the corresponding positive equilibrium pair is absent, whereas for $c>c_{c}$ two positive equilibrium branches are observed. The leading Jacobian eigenvalue approaches zero as $c\to c_{c}$, providing numerical evidence of the predicted fold transition. Gillespie simulations are additionally performed on both sides of $c_{c}$ to illustrate the corresponding stochastic population behavior.

### 5.9. Interpretation for the Stochastic Model

Because the original stochastic process has a finite state space and an absorbing extinction state, a positive deterministic equilibrium should not be interpreted as a nontrivial stationary distribution of the exact CTMC. Instead, it represents a long-lived dynamical state around which the stochastic process may fluctuate before eventual extinction. The fold transition is therefore not interpreted as a literal bifurcation of the finite-state stochastic process. Its stochastic counterpart is manifested indirectly through changes in transient and quasi-stationary population behavior near the deterministic critical capacity. In particular, the Gillespie simulations around $c_{c}$ quantify finite-horizon survival, conditional bacterial burden, and resistant fraction, providing a stochastic assessment of the population behavior near the deterministic transition.

Accordingly, near a stable positive equilibrium, useful stochastic quantities include the extinction time, finite-horizon survival probability, conditional bacterial burden given survival, and the distribution of the population state before extinction. These quantities can be estimated directly using Monte Carlo simulation.

The critical transition identified from the deterministic equations therefore serves as a structural guide for the stochastic analysis rather than as a claim of an infinite stationary bacterial load. In particular, the finite carrying capacity *K* prevents an actual divergence of the population size, while the absorbing state ensures that the exact process ultimately reaches extinction with probability one.

The stochastic consequences of the equilibrium transitions, including extinction probabilities, extinction times, conditional bacterial burden, and uncertainty quantification, are investigated numerically in Section 6.

## 6. Monte Carlo Simulation and Numerical Validation

The stochastic component of the analysis is complemented by Monte Carlo simulation using the Gillespie direct method [17]. The purpose of the simulation study is not to estimate an ordinary stationary distribution, since the finite-state process has an absorbing extinction state. Instead, the simulations are used to investigate finite-time extinction, survival, bacterial burden conditional on survival, and the stochastic manifestation of the equilibrium transitions identified in Section 4 and Section 5.

The simulation study is therefore organized around three objectives: (i) Verification of the stochastic transition mechanism; (ii) Numerical assessment of the deterministic equilibrium structure; (iii) Quantification of extinction and persistence behavior under different treatment and mutation scenarios.

### 6.1. Gillespie Simulation Algorithm

Let the current state of the process be

$$N\left(t\right)=(N_{s}\left(t\right),N_{r}\left(t\right)).\;$$

For a state $\left(n_{s},n_{r}\right)$, the five event rates are the transition rates already defined in Equations (2)–(6), namely

$$b_{s}\left(n_{s},n_{r}\right),\;b_{r}\left(n_{s},n_{r}\right),\;d_{s}\left(n_{s},n_{r}\right),\;d_{r}\left(n_{s},n_{r}\right),\;m\left(n_{s},n_{r}\right).\;$$

The total event rate is therefore

(75) $$a_{0}\left(n_{s},n_{r}\right)=b_{s}\left(n_{s},n_{r}\right)+b_{r}\left(n_{s},n_{r}\right)+d_{s}\left(n_{s},n_{r}\right)+d_{r}\left(n_{s},n_{r}\right)+m\left(n_{s},n_{r}\right).$$

Provided that $a_{0}\left(n_{s},n_{r}\right)>0$, the waiting time until the next event is generated according to

(76) $$\Delta t∼\mathrm{Exp}\left(a_{0}\left(n_{s},n_{r}\right)\right).$$

The next event is selected from the five transition mechanisms with probabilities

$$\frac{b_{s}}{a_{0}},\;\frac{b_{r}}{a_{0}},\;\frac{d_{s}}{a_{0}},\;\frac{d_{r}}{a_{0}},\;\frac{m}{a_{0}}.\;$$

A death event is possible only when the corresponding population is positive. The simulation terminates when

$$N_{s}\left(t\right)+N_{r}\left(t\right)=0,$$

or when the prescribed observation horizon *T* is reached.

### 6.2. Initial Conditions and Parameter Design

Unless otherwise specified, simulations are initialized with

(77) $$N_{s}\left(0\right)=100,\;N_{r}\left(0\right)=0.\;$$

This initial condition represents an infection dominated by the antibiotic-sensitive population with no initially detectable resistant subpopulation. Resistant bacteria may subsequently arise through the mutation mechanism at rate $\nu N_{s}\left(t\right)$. The baseline parameter values are selected as

(78) μ=1,ϵ=0.1,c=5,

with

$$K\in \{10^{3},10^{4}\}.$$

The sensitive growth rate $\lambda_{s}$, resistant growth rate $\lambda_{r}$, and mutation rate ν are varied systematically. When a fitness cost of resistance is considered, we use

(79) $$\lambda_{r}=\eta \lambda_{s},\;0<\eta <1,\;$$

with η=0.8 used as a representative value. Importantly, parameter combinations are not selected according to an effective traffic intensity. Instead, they are selected according to the local stability conditions and the positive-equilibrium structure derived in Section 3, Section 4 and Section 5. In particular, simulations are performed in the following qualitatively different regimes:

1. Parameters for which the extinction state is locally stable and no admissible positive equilibrium is detected;
2. Parameters for which the extinction state is locally stable but a stable positive equilibrium coexists with it;
3. Parameters close to a capacity-induced critical point;
4. Parameters for which the positive equilibrium has disappeared following a fold transition.

This design allows the stochastic simulations to test and validate the deterministic equilibrium structure across distinct parameter regimes, including extinction, persistence, and capacity-induced transitions.

### 6.3. Simulation Replications

For each parameter combination, we generate

$$M=10^{4}\;$$

independent stochastic trajectories. The maximum simulation time is denoted by $T_{\max}$.

For each trajectory *m*, the extinction time is defined as

(80) $$\tau_{0}^{\left(m\right)}=\inf\left\{t\geq 0:N_{s}^{\left(m\right)}\left(t\right)+N_{r}^{\left(m\right)}\left(t\right)=0\right\}.$$

If extinction occurs before $T_{\max}$, the exact extinction time is recorded. If extinction does not occur before $T_{\max}$, the trajectory is classified as surviving over the observation horizon. This distinction is essential because a trajectory that survives until $T_{\max}$ should not be interpreted as having reached a stationary state.

### 6.4. Finite-Horizon Survival Probability

For a prescribed observation time *T*, define the indicator

(81) $$I_{m}\left(T\right)=1\left\{\tau_{0}^{\left(m\right)}>T\right\}.$$

The finite-horizon survival probability is estimated by

(82) $$\hat{S}\left(T\right)=\frac{1}{M}\sum_{m=1}^{M}I_{m}\left(T\right).$$

Because the trajectories are independent, the Monte Carlo standard error is

(83) $$\mathrm{SE}(\hat{S}\left(T\right))=\sqrt{\frac{\hat{S}\left(T\right)\left[1-\hat{S}\left(T\right)\right]}{M}}.$$

A corresponding approximate 95% confidence interval is

(84) $$\hat{S}\left(T\right)\pm 1.96\;\mathrm{SE}(\hat{S}\left(T\right)).$$

For probabilities close to zero or one, a binomial interval such as the Wilson score interval can be used instead of the normal approximation. The Wilson score interval is a confidence interval for a binomial proportion that provides improved coverage, particularly when the estimated proportion is close to zero or one.

### 6.5. Extinction-Time Analysis

The distribution of extinction times provides a direct stochastic measure of treatment effectiveness. For trajectories that become extinct before $T_{\max}$, the empirical mean extinction time is

(85) $${\hat{\tau }}_{0}=\frac{1}{M_{E}}\sum_{m:\tau_{0}^{\left(m\right)}\leq T_{\max}}\tau_{0}^{\left(m\right)},$$

where $M_{E}$ is the number of trajectories that reach extinction within the observation window. Thus, ${\hat{\tau }}_{0}$ estimates the conditional mean extinction time

$$E\;\left(\tau_{0}|\tau_{0}\leq T_{\max}\right),$$

rather than the unconditional mean extinction time, because trajectories that survive beyond $T_{\max}$ are right-censored and do not contribute an observed extinction time.The empirical survival function of the extinction time is estimated by

(86) $${\hat{S}}_{\tau }\left(t\right)=\frac{1}{M}\sum_{m=1}^{M}1\left\{\tau_{0}^{\left(m\right)}>t\right\}.$$

This quantity is particularly useful near a critical transition, where trajectories may exhibit long-lived excursions before extinction. Because simulations may be right-censored at $T_{\max}$, the extinction-time distribution should not be summarized only by the arithmetic mean when a substantial proportion of trajectories survive beyond the observation window. In such cases, the empirical survival curve or a Kaplan–Meier estimate provide a more appropriate summary.

### 6.6. Conditional Bacterial Burden

Since the exact stochastic process eventually reaches extinction, an unconditional long-run mean bacterial population is not an appropriate performance measure. Instead, for a finite observation time *T*, define the total bacterial burden by

(87) $$N\left(t\right)=N_{s}\left(t\right)+N_{r}\left(t\right).$$

The conditional mean bacterial burden among trajectories that survive to time *T* is

(88) $$\hat{B}\left(T\right)=\frac{\sum_{m=1}^{M}N^{\left(m\right)}\left(T\right)I_{m}\left(T\right)}{\sum_{m=1}^{M}I_{m}\left(T\right)}.$$

Thus, $\hat{B}\left(T\right)$ estimates the conditional mean bacterial burden

$$E\;\left[N\left(T\right)|\tau_{0}>T\right],$$

where the conditioning restricts the calculation to trajectories that remain non-extinct at time *T*. Similarly, the conditional resistant fraction is

(89) $${\hat{P}}_{r}\left(T\right)=\frac{\sum_{m=1}^{M}N_{r}^{\left(m\right)}\left(T\right)I_{m}\left(T\right)}{\sum_{m=1}^{M}N^{\left(m\right)}\left(T\right)I_{m}\left(T\right)},$$

provided that the denominator is positive. These conditional quantities describe the population burden among infections that have not yet been eradicated and therefore provide a more meaningful measure of treatment performance than an ordinary stationary mean.

### 6.7. Time-Integrated Bacterial Burden

To quantify cumulative bacterial exposure over a finite treatment interval, we also define the integrated burden

(90) $$A_{T}^{\left(m\right)}=\int_{0}^{T\wedge \tau_{0}^{\left(m\right)}}N^{\left(m\right)}\left(t\right)\;dt.$$

The Monte Carlo estimate is

(91) $${\hat{A}}_{T}=\frac{1}{M}\sum_{m=1}^{M}A_{T}^{\left(m\right)}.$$

The corresponding resistant burden is

(92) $$A_{r,T}^{\left(m\right)}=\int_{0}^{T\wedge \tau_{0}^{\left(m\right)}}N_{r}^{\left(m\right)}\left(t\right)\;dt.$$

The ratio

(93) $$R_{T}=\frac{\sum_{m=1}^{M}A_{r,T}^{\left(m\right)}}{\sum_{m=1}^{M}A_{T}^{\left(m\right)}}$$

provides a finite-horizon measure of the contribution of resistant bacteria to the cumulative bacterial burden.

### 6.8. Numerical Verification of Deterministic Equilibria

The deterministic approximation developed in Section 4 provides candidate equilibria $\left(x_{s}^{*},x_{r}^{*}\right)$. These predictions are compared with stochastic simulation at sufficiently large population sizes. For a parameter combination with a stable positive equilibrium, the simulation distribution at time *T* is examined conditional on survival, and its empirical mean is compared with

$$(x_{s}^{*},x_{r}^{*}).$$

Define

(94) $$\hat{x}\left(T\right)=\left(\begin{array}{c} {\hat{x}}_{s}\left(T\right)\\ {\hat{x}}_{r}\left(T\right) \end{array}\right),$$

where

(95) $${\hat{x}}_{s}\left(T\right)=\frac{\sum_{m=1}^{M}N_{s}^{\left(m\right)}\left(T\right)I_{m}\left(T\right)}{\sum_{m=1}^{M}I_{m}\left(T\right)},$$

(96) $${\hat{x}}_{r}\left(T\right)=\frac{\sum_{m=1}^{M}N_{r}^{\left(m\right)}\left(T\right)I_{m}\left(T\right)}{\sum_{m=1}^{M}I_{m}\left(T\right)}.$$

The relative equilibrium discrepancy is quantified by

(97) $$E_{\mathrm{eq}}=\frac{{∥\hat{x}\left(T\right)-x^{*}∥}_{2}}{{∥x^{*}∥}_{2}}.$$

The observation time *T* is chosen to be sufficiently large for the surviving trajectories to approach the corresponding long-lived population regime, while avoiding interpretation of the resulting distribution as an ordinary stationary distribution.

### 6.9. Numerical Detection of the Critical Transition

For a selected control parameter θ, the deterministic equilibrium equations are solved over a grid

$$\theta_{1},\theta_{2},\ldots ,\theta_{J}.$$

At each value of θ, all biologically admissible positive equilibria are identified and their Jacobian eigenvalues are calculated. A candidate critical parameter $\theta_{c}$ is identified when

(98) $$\det J(x^{*};\theta_{c})\approx 0$$

and one eigenvalue approaches zero. The stochastic simulation is then performed on both sides of the candidate critical value. The following quantities are examined:

(99) $$\hat{S}\left(T\right),\;{\hat{\tau }}_{0},\;\hat{B}\left(T\right),\;{\hat{A}}_{T}.$$

A sharp change in these quantities near $\theta_{c}$ provides numerical evidence for the stochastic manifestation of the deterministic transition.

### 6.10. Representative Monte Carlo Results

To illustrate the stochastic consequences of the analytical classification, we consider three representative parameter regimes. All simulations use

$$\mu =1,\;\epsilon =0.1,\;c=5,\;K=10^{3},$$

with

$$N_{s}\left(0\right)=100,\;N_{r}\left(0\right)=0,\;T_{\max}=100,$$

and $M=10^{4}$ independent Gillespie trajectories.

The first regime is extinction-dominated, with both local growth conditions unfavorable to bacterial persistence. The second regime has a resistant population capable of local invasion because $\lambda_{r}>\epsilon \mu$, despite the absence of initial resistant bacteria. The third regime admits a stable positive deterministic equilibrium, and the initial population lies in its long-lived basin of attraction. The corresponding Monte Carlo results are reported in Table 2.

For the extinction-dominated regime, only a small fraction of trajectories survive to the observation time, and the surviving population is predominantly resistant. In the resistant-invasion regime, approximately one third of the trajectories remain non-extinct at T=100, with the conditional population being almost entirely resistant. In contrast, the long-lived coexistence regime produces a bacterial burden close to the stable deterministic equilibrium.

For the third regime, the deterministic equilibrium is approximately

$$\left(x_{s}^{*},x_{r}^{*}\right)=\left(996.16,0.996\right),\;$$

whereas the corresponding conditional Monte Carlo estimates are

$$\left({\hat{x}}_{s},{\hat{x}}_{r}\right)=\left(996.02,1.01\right).\;$$

Thus, the stochastic simulation provides a numerical illustration of consistency with the deterministic equilibrium approximation while maintaining the distinction between a long-lived stochastic regime and an ordinary stationary distribution.

### 6.11. Mutation-Rate Experiments

To quantify the effect of mutation, we systematically vary

$$\nu \in \{0,\;2.5\times 10^{-4},\;5\times 10^{-4},\;10^{-3},\;1.5\times 10^{-3},\;2\times 10^{-3}\},\;$$

while keeping the remaining parameters fixed. For each value of ν, we calculate the finite-horizon survival probability $\hat{S}\left(T;\nu \right)$, the conditional bacterial burden $\hat{B}\left(T;\nu \right)$, the conditional resistant fraction ${\hat{P}}_{r}\left(T;\nu \right)$, and the cumulative bacterial burden ${\hat{A}}_{T}\left(\nu \right)$. The results show the progressive effect of mutation on the phenotypic composition of the surviving bacterial population. Increasing ν transfers a larger fraction of the sensitive population into the resistant class and therefore changes the relative contribution of resistant bacteria to the total burden.

### 6.12. Effect of Treatment Capacity

To quantify the effect of treatment capacity, we systematically vary

c∈{50,75,100,125,130,135,150,200}.

For each value of *c*, the admissible deterministic equilibria and their stability are recalculated, followed by Gillespie simulations using the same initial condition and observation horizon. The quantities

$$\hat{S}\left(T;c\right),\;\hat{B}\left(T;c\right),\;{\hat{\tau }}_{0}\left(c\right),\;{\hat{A}}_{T}\left(c\right)$$

are then evaluated. The results show the progressive effect of mutation on the phenotypic composition of the surviving bacterial population. Increasing ν transfers a larger fraction of the sensitive population into the resistant class and therefore changes the relative contribution of resistant bacteria to the total burden.

## 7. Conclusions

This study developed a finite-state stochastic population model for antibiotic-sensitive and antibiotic-resistant bacteria under capacity-limited treatment. The model integrates density-dependent growth, differential treatment susceptibility, mutation, and finite treatment capacity within a continuous-time Markov chain framework. The analysis distinguishes eventual stochastic extinction from long-lived non-extinct behavior and identifies separate sensitive and resistant growth conditions. The deterministic equilibrium analysis further shows that capacity-induced nondegenerate folds are associated with square-root equilibrium scaling and critical slowing down. Gillespie-based Monte Carlo simulations illustrate how these deterministic mechanisms are reflected in finite-time survival, extinction, and conditional bacterial burden. Overall, the framework provides a unified mathematical description of the interaction between antibiotic resistance and capacity-limited treatment while maintaining the distinction between deterministic equilibrium behavior and eventual stochastic extinction.

## Figures and Tables

**Table 1:** Gillespie simulation results near the critical treatment capacity. The reported intervals are approximate 95% confidence intervals.

*c*	$\hat{S}\left(100\right)$	$\hat{B}\left(100\right)$	${\hat{P}}_{r}\left(100\right)$
129.0000	100.00%	9707.28±0.35	0.35046±0.00010
129.5723	100.00%	9705.75±0.35	0.35005±0.00010
130.0000	100.00%	9705.13±0.35	0.34970±0.00010

**Table 2:** Representative Monte Carlo results for the sensitive–resistant bacterial population model. Survival is evaluated at T=100. The conditional means are calculated only among trajectories surviving until *T*. The mean extinction time is calculated only for trajectories that become extinct before $T_{\max}$. A dash indicates that no extinction time is reported because no trajectory became extinct before $T_{\max}$.

Scenario	$\lambda_{s}$	$\lambda_{r}$	ν	$\hat{S}$(100)	${\hat{\tau }}_{0}$	${\hat{N}}_{s}$(100)	${\hat{N}}_{r}$(100)
Extinction-dominated	0.01	0.05	0.001	0.68%	28.47	0.00	39.26
Resistant invasion	0.01	0.15	0.001	32.65%	25.27	0.00	930.33
Long-lived coexistence	1.80	0.01	0.0001	100.00%	–	996.02	1.01

## Data Availability

The original contributions presented in this study are included in the article. Further inquiries can be directed to the corresponding author.
